# Waldenström Macroglobulinemia: Clinical and Immunological Aspects, Natural History, Cell of Origin, and Emerging Mouse Models

**DOI:** 10.1155/2013/815325

**Published:** 2013-09-09

**Authors:** Siegfried Janz

**Affiliations:** Department of Pathology, University of Iowa Carver College of Medicine, 1030 ML, Iowa City, IA 52242, USA

## Abstract

Waldenström macroglobulinemia (WM) is a rare and currently incurable neoplasm of IgM-expressing B-lymphocytes that is characterized by the occurrence of a monoclonal IgM (mIgM) paraprotein in blood serum and the infiltration of the hematopoietic bone marrow with malignant lymphoplasmacytic cells. The symptoms of patients with WM can be attributed to the extent and tissue sites of tumor cell infiltration and the magnitude and immunological specificity of the paraprotein. WM presents fascinating clues on neoplastic B-cell development, including the recent discovery of a specific gain-of-function mutation in the MYD88 adapter protein. This not only provides an intriguing link to new findings that natural effector IgM^+^IgD^+^ memory B-cells are dependent on MYD88 signaling, but also supports the hypothesis that WM derives from primitive, innate-like B-cells, such as marginal zone and B1 B-cells. Following a brief review of the clinical aspects and natural history of WM, this review discusses the thorny issue of WM's cell of origin in greater depth. Also included are emerging, genetically engineered mouse models of human WM that may enhance our understanding of the biologic and genetic underpinnings of the disease and facilitate the design and testing of new approaches to treat and prevent WM more effectively.

## 1. Clinical Aspects of WM: A Brief Overview

### 1.1. Definition and Classification

The 2008 World Health Organization (WHO) Classification of Tumours of Haematopoietic and Lymphoid Tissues [1] defines Waldenström macroglobulinemia (WM) as a type of lymphoplasmacytic lymphoma (LPL) that involves the bone marrow and is associated with a monoclonal immunoglobulin (Ig) of the M class in the serum. The monoclonal IgM is usually referred to as IgM paraprotein or “M spike”—or mIgM for short. LPL is a low-grade malignancy of the mature B-lymphocyte lineage that exhibits a cytological spectrum of lymphoplasmacytic differentiation that ranges from small B cells to fully differentiated plasma cells (PCs). Between these extremes lies a sizable, if not predominant, fraction of cells with intermediate features and, therefore, designated lymphoplasmacytoid or lymphoplasmacytic cells (LPCs) [2]. Sometimes these cells are referred to as plasmacytoid or plasmacytic lymphocytes. Although LPL is characteristically associated with an mIgM that can be readily detected by serum protein electrophoresis, LPL does not always lead to WM. This is because approximately 5% of LPLs either produce a paraprotein that is not of the M class (but instead belongs in most cases to the A class or one of the four G subclasses) or do not produce paraprotein at all (nonsecretory variant). Similarly, LPL is not the sole underlying cause of a serum IgM spike, because paraproteins of this sort can also be produced by other types of B cell lymphoma with plasmacytic differentiation potential (e.g., marginal zone B cell lymphoma, MZL) [3] or, in rare cases, by *bona fide* plasma cell neoplasms, such as IgM^+^ plasmacytoma or multiple myeloma (MM) [4]. In sum, even though LPL does not always lead to WM and the occurrence of a serum IgM spike is not pathognomonic for this disease, WM is always caused by IgM^+^ LPL.

### 1.2. Symptoms Attributable to Tumor Growth

The great majority of patients with LPL exhibit distinctive clinical features that can be attributed either to tissue infiltration with malignant B cells or IgM-dependent changes in serum (hyperviscosity syndrome) and/or various tissue sites (immunoglobulin deposition disease, autoimmunity). With regard to tissue infiltration by tumor cells, the replacement of the normal hematopoietic bone marrow with WM cells usually leads to a progressive normochromic or normocytic anemia and, to a lesser extent, suppression of other blood cell lineages leading, for example, to thrombocytopenia. Tumor infiltrates in solid tissues may clinically present as organomegalies, including hepato- and splenomegaly as well as lymphadenopathy. In rare cases, malignant infiltration of the lung (accompanied by pleural effusion) [5], the gastrointestinal tract [6], and the skull (involving the orbitae [7] or generating epidural masses) has been observed. Bing-Neel syndrome—which consists of headache, vertigo, impaired hearing, ataxia, nystagmus, diplopia, and, in terminal stages, coma—is a vicious CNS (central nervous system) complication of WM caused by blood vessel damage, IgM deposition, and perivascular lymphoma cell infiltration in the brain and spinal nerves [8]. Malignant vitritis and conjunctival infiltration are rare ocular manifestations of WM. The syndromic presentation of IgM paraproteinemia and associated clinical features was first recognized by the Swedish doctor of internal medicine, Jan Gösta Waldenström, who published his initial observations in the 1940s. His findings were swiftly embraced by hematologists in other countries and, within a few years, the term Waldenström macroglobulinemia was coined and commonly accepted. Since Waldenström's landmark report some 70 odd years ago, we have learned a great deal about the clinical presentations and complications of the disease, including the symptoms attributable to the hallmark IgM monoclonal gammopathy, which will be described in the following.

### 1.3. Symptoms Attributable to mIgM

Under normal conditions, IgM predominantly occurs in serum in pentameric form. Five IgM monomers, each consisting of two *μ* heavy chains and two *κ* or *λ* light chains, are covalently linked by the J or joining chain, resulting in a supramolecular complex that is often schematically depicted as a snow flake or five-leafed shamrock. The pentameric structure of IgM results in a large molecular mass (~970 kilodalton), high avidity to antigen (10 antigen-binding sites), and high potential for complement activation, but the flip side of these features is poor diffusion properties, low concentration in interstitial fluids, and poor ability to leave the blood stream. In patients with WM, the elevated concentration of monoclonal IgM can lead to serum hyperviscosity, a key distinguishing feature of the disease. Symptoms include bleeding and a multitude of ocular, neurologic, and cardiovascular manifestations [9]. Thanks to an earlier recognition of the disease in recent years, serum hyperviscosity is only observed in a minority of patients at diagnosis [10, 11]. As a rule, symptoms of hyperviscosity are rare in patients with IgM levels below 30–40 g/L and serum viscosity values below 4 cP (centipoise). This threshold corresponds to a 2.5-fold increase relative to normal serum viscosity, which is in the neighborhood of 1.6 cP. Some symptoms of WM are attributable to tissue deposition of IgM, not to increased serum viscosity. IgM deposits may occur in kidney (glomeruli), intestine, and skin, leading to proteinuria, diarrhea, and characteristic papules (IgM storage papules or cutaneous macroglobulinosis) [12, 13], respectively. Kidney involvement usually leads to slowly progressive loss of function rather than acute renal failure [14, 15]. Primary amyloidosis due to monoclonal light chain deposition has been found, in descending order of frequency, in the heart, peripheral nerves, kidneys, soft tissues, liver, and lungs [16]. In contrast, secondary (reactive) amyloidosis is rarely seen [17]. WM patients may also exhibit symptoms that are attributed to autoantibody activity of IgM. IgM*κ* with specificity to certain red blood cell antigens may lead to chronic immune hemolytic anemia that is associated with elevated cold agglutinin titers [18, 19]. The combination of mIgM, urticaria, fever, and arthralgia is known as Schnitzler syndrome [20]. Neuropathies, which are caused in part by immunoreactivity of IgM to myelin-associated glycoprotein (MAG) [21], IgM-mediated glomerulonephritis, angioedema, and acquired von Willebrand disease have all been reported. A laboratory finding without adverse health effects in approximately one fifth of patients with WM is the propensity of IgM to undergo precipitation at temperatures below normal body temperature; for example, during storage of serum at 4°C in a refrigerator. However, in a small subset of cases (<5%), this phenomenon, known as cryoglobulinemia, causes symptoms including Raynaud syndrome, joint pain, and purpura and other skin changes [22].

### 1.4. Differential Diagnosis

As stated in the introductory section entitled “definition and classification,” neither the presence of a malignant LPC clone in bone marrow nor the detection of an IgM spike in serum is pathognomonic for WM. Instead, these changes may also be caused by other IgM^+^ malignancies of the mature B cell lineage related to but distinct from WM. Splenic marginal zone lymphoma (SMZL) appears to be a particular concern and sometimes a challenge for diagnosticians, yet mantle cell lymphoma (MCL), rare cases of IgM-producing multiple myeloma (IgM-MM), and B cell chronic lymphocytic leukemia (B-CLL) must also be considered when a patient presents with symptoms suggestive of WM. SMZL can be distinguished from WM on the basis of immunophenotypic and molecular cytogenetic findings: CD11c (integrin alpha X chain) is more highly expressed in patients with SMZL, whereas CD25 (IL-2 receptor *α* chain) is twice as common in WM [23]. CD103, a member of the integrin adhesion surface receptor family of protein, is invariably absent on WM cells but detected in 40% of patients with SMZL [23]. The genomic abnormality most common in SMZL, loss of 7q31-32 [24], is not seen in WM. Additionally, SMZL exhibits a specific gene expression signature upon microarray-based, genome-wide analysis of the tumor transcriptome [25, 26]. MCL can be distinguished from WM based on clinicohistopathologic findings [27] and, in terms of cancer cytogenetics, the almost invariable occurrence of the hallmark t(11;14)(q13;q32) translocation that recombines *IGH* (Ig heavy-chain locus) on chromosome 14 and *CCND1* (cyclin D1) on chromosome 11 [28, 29]. IgM^+^ myeloma can be distinguished from WM by virtue of its pronounced plasma cell morphology and presence of lytic bone lesions. Renal insufficiency is more common in IgM-MM than in WM. Chromosomal translocations involving *IGH*, particularly the cyclin D1-activating t(11;14)(q13;q32) exchange mentioned previously, occur in IgM-MM but not WM [30, 31]. B-CLL may mimic WM clinically, but the morphological and immunophenotypic features of the tumor cells are usually sufficiently different from WM to avoid confusion. CLL is positive for CD5 (surface protein that mitigates activating signals from the B cell receptor) and CD23 (low-affinity IgE receptor) by flow cytometry. Another area of concern from a diagnostic and clinical management point of view is the possibility that the low-grade lymphoma, WM, progresses to the high-grade lymphoma, diffuse large B cell lymphoma (DLBCL). This is usually associated with aggressive clinical course, profound cytopenias, extramedullary disease, and poor outcome [32]. The potentially diverse nature of “histological transformation events” in patients with WM, including those involving EBV (Epstein-Barr virus) infection, is increasingly recognized [32]. 

### 1.5. Risk Stratification and Prognosis

Owing to novel agents and combination therapies briefly discussed in the following section, overall survival of WM patients of all ages has improved [33–35]. Nonetheless, WM remains an incurable disease that exhibits significant variations in clinical course and outcome [36]. The latter may depend in part on differences between patient cohorts in different studies (e.g., tumor burden at presentation) but may also be a consequence of differences among individual tumors with respect to genetic, epigenetic, and biological determinants of tumor growth and IgM production [37–40]. The recently updated International Prognostic Scoring System for WM [41] uses five determinants for disease staging: age (above 65 is a negative risk factor), hemoglobin (≤115 g/L), platelets (≤10^5^ per microliter), *β*2 microglobulin (>3 mg/L), and mIgM (>70 g/L). After assessing one point for each negative risk factor, the patient can be categorized as low risk (≤1 except age), intermediate risk (2 or older than 65 yrs) or high risk (>2). The 3 risk groups are associated with a median survival of more than 10 years (143 months), 8 years (99 months), and 3.5 years (44 months), respectively. Age has a profound impact on risk stratification and prognosis as, by definition, patients older than 65 years cannot be assigned to the low-risk category regardless how subdued the disease might be. In comparison to age, IgM levels are weighed more forgivingly, as this parameter does not enter the staging system until a threshold of 70 g/L is exceeded. This is perhaps somewhat of a surprise given that mIgM levels correlate with abundance of monotypic plasma cells in bone marrow [42], a finding that many may have intuitively considered a marker of disease progression. Ongoing efforts to refine the staging system focus on serum levels of lactate dehydrogenase, which have been recently shown to stratify high-risk patients into two subgroups with significantly different outcomes [43, 44], and the immunoglobulin free light-chain assay, which is under review as a potential prognosticator of patients with WM [45]. Importantly, the prognostic scoring system for WM should not be used for treatment decisions. This is the prerogative of clinicians, who will base the determination whether a patient requires treatment or not on the available clinical and laboratory findings, as well as the preferences of the patient with WM. With regard to overall outcome of WM, it is important to realize that WM takes an indolent course and patients with WM usually are of an advanced age. In fact, nearly half of them succumb to diseases of the elderly population—unrelated to WM [11]. This led to the introduction of cause-specific survival as an important outcome measure for patients with WM, which censors (disregards) patients who die of causes other than WM [46]. 

### 1.6. Treatment

Treatment is reserved for symptomatic patients. It usually consists of alkylating agents (chlorambucil, cyclophosphamide, and melphalan), nucleoside analogues (cladribine and fludarabine), proteasome inhibitors (bortezomib), dexamethasone, and monoclonal antibody to CD20 (rituximab). Regimens for frontline and salvage treatments, using the after mentioned drugs either alone (monotherapy) or in combination, have been established and expertly reviewed by leading WM clinicians [11, 47–50]. Therapeutic options involving drugs can be supplemented with hematopoietic stem cell (HSC) transplantation, particularly with the autologous version, which appears to be highly effective but underutilized [51]. 

A recently completed phase 3 trial in patients with WM, which is thought to have defined a new benchmark for future clinical studies on the disease, demonstrated that fludarabine monotherapy was more effective than chlorambucil in terms of progression-free survival (37.8 versus 27.1 months), duration of response (38.5 versus 21.3 months), and overall survival (median not reached versus 69.8 months). The results suggested that fludarabine may be the drug of choice for frontline treatment of patients that are not candidates for autologous HSC transplants but have poor prognostic factors and/or are in need of rapid disease control [52]. Although the clinical trial (an area of research that is notorious for lack of well-designed phase 3 trials) reported interesting findings and was well received, it may not lend itself to defining a new standard of care because of inadvertent shortcomings [53]. For example, the trial's treatment plan did not incorporate rituximab, even though this therapeutic antibody has become a mainstay in the care for patients with WM [54–56].

Additional drugs that may be useful for patients with WM include monoclonal antibody to CD52 (alemtuzumab) [57], immunomodulatory agents (thalidomide [58] and lenalidomide [59]), and molecularly targeted small-molecule inhibitors of cellular signal transduction pathways, such as the mTOR (mammalian target of rapamycin) inhibitor, everolimus [60], the AKT (v-akt murine thymoma viral oncogene homolog 1) inhibitor, perifosine [61], and newly emerging HDAC (histone deacetylase) inhibitors [62]. A simple and almost forgotten drug, the nitrogen mustard-related alkylating agent bendamustine—which had been developed as early as 1963 in the former East Germany, where it remained dormant and unavailable to the Western world until reunification of Germany in 1990—has recently experienced a renaissance in the treatment of WM and related low-grade lymphomas [63, 64]. The management of IgM-dependent hyperviscosity syndrome (HVS) involves plasmapheresis, which is able to acutely rid the patient serum of the abnormal immunoglobulin, is safe and effective, and is usually well tolerated [65]. Randomized, controlled clinical trials for treatment of serum HSV are lacking, but plasmapheresis is widely accepted as an effective short-term treatment for patients with WM [66]. A very recent, interesting development in the treatment of WM-dependent anemia is parental iron administration [67].

### 1.7. Key Points and Future Directions

**Figure 1:**
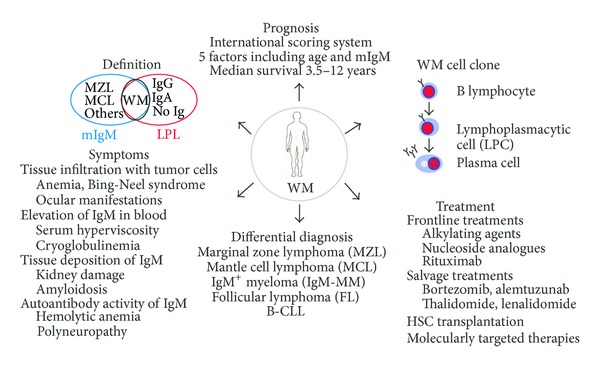
Synopsis of clinicopathological features and treatment of WM. See Section 1 for details.

A synopsis of salient points covered in this chapter is schematically presented in Figure 1. WM is a low-grade blood cancer of the mature B-lymphocyte lineage that is closely related to, but distinct from, other types of low-grade B cell lymphoma, such as SMZL, MCL, and B-CLL. The features of WM and its differential diagnosis are well established. Treatment of WM is highly effective, and long-term control with good clinical management is possible. Nevertheless, WM remains an incurable neoplasm at this juncture. Experienced WM clinicians increasingly emphasize that the long survival and advanced age of the great majority of patients with WM call for greater awareness and consideration of quality-of-life and treatment-associated morbidity issues. Selecting the most appropriate interventions for patients with WM and managing the complications of progressive disease remain ongoing tasks. Expert reviews providing tons of valuable information about clinical aspects of WM are available [48, 50, 68, 69].

## 2. Epidemiology and Natural History of WM

### 2.1. Incidence

With an annual overall incidence rate of approximately 3 cases per one million persons, WM accounts for a little less than 2% of non-Hodgkin lymphomas in the United States. This number corresponds to approximately 1,500 new cases per year in the US [70]. Incidence in men and Caucasians is higher than in woman and African Americans, respectively [71, 72]. The median age at diagnosis is approximately 70 years [73]. The strongest risk factor for WM is IgM MGUS, the “premalignant” precursor condition of WM. On average, IgM MGUS progresses to frank WM (or one of its relatives among the B cell proliferative disorders) at an annual rate of 1.5% [74, 75]. This means that in a hypothetical cohort of 150 individuals with IgM MGUS (the assembly of which is not an easy task to begin with) only one individual per annum will go on to develop WM. Obviously, the slow progression rate causes great difficulties for designing sufficiently powered intervention trials; for example, those aimed at evaluating whether chemoprevention can block the IgM MGUS to WM transition. Trials along this line may take decades to produce meaningful statistical results. This backdrop provides a strong rationale for developing experimental model systems of WM, with which novel tumor prevention approaches can be evaluated in a reasonable time frame and at reasonable costs. Model systems of this sort may take advantage of nonmammalian organisms, such as round worm (*Caenorhabditis elegans*), fruit fly (*Drosophila melanogaster*), zebra fish (*Danio rerio*), or frog (*Xenopus laevis, tropicalis*), or of mammals, such as rat, rabbit, dog, pig, and others. Model organisms of human cancer are widely available in research laboratories around the world and have been used extensively to shed light on mechanisms of tumor development and progression. Chapter 6 of this spotlight paper will argue that accurate, genetically engineered mouse models of human WM may be particularly useful for designing and testing new prevention approaches to WM. 

### 2.2. Etiology

The etiology of WM is unknown. Recent research advances, owing in large measure to four studies summarized in the following, have all implicated autoimmune and chronic inflammatory conditions in the causation of the disease. The studies involved hundreds of thousands of individuals and arrived at the same conclusion despite different study designs. Two studies relied on surveys and were performed in the US. Two studies were population based and carried out in Sweden. The following observations were made. Two nationwide surveys of US veterans explored the role of antigen stimulation in the pathogenesis of WM. The first survey, which included 146,394 individuals infected with hepatitis C virus (HCV) and 572,293 controls, revealed that HCV infection increased the risk of developing WM approximately 3-fold [76]. The second survey, which identified 361 patients with WM among 4 million U.S. veterans followed for up to 27 years, found a ~2.5-fold elevated risk of WM in individuals with a personal history of an autoimmune disease and a smaller but still notably elevated risk conferred by hepatitis, rickettsiosis, and infection with human immunodeficiency virus (HIV) [77]. The Swedish studies produced similar results. The first one evaluated 2,470 patients with LPL/WM and 9,698 matched controls, as well as almost 30,000 first-degree relatives of the patients and controls. It uncovered an association of LPL/WM with a personal history of autoimmune diseases (e.g., Sjögren syndrome, hemolytic anemia, polymyalgia rheumatica, and giant cell arteritis) and infectious diseases (e.g., pneumonia, pyelonephritis, sinusitis, herpes zoster, and influenza). Additionally, it revealed a link of LPL/WM with a family history of Sjögren syndrome, autoimmune hemolytic anemia, Guillain-Barré syndrome, cytomegalovirus, gingivitis/periodontitis, and chronic prostatitis [78]. The second study assessed 5,403 patients with MGUS and 21,209 matched controls, together with their respective first-degree relatives. It showed that personal and family history of autoimmune disease were independently associated with increased risk of MGUS. It also linked a personal history of infection and chronic inflammation (but not a family history) to increased risk of MGUS [79]. These findings furthered our understanding of the underlying cause of WM by implicating sustained B cell stimulation with self and/or exogenous antigen in the pathophysiology of the disease. 

### 2.3. Genetic Predisposition

Familial clustering of LPL/WM, which has been documented repeatedly in independent studies going back to the 1980s [80–83], points to inherited traits or “WM alleles” that predispose affected individuals to IgM MGUS and WM. The findings indicated that WM alleles segregate in extended families that exhibit genetic proclivity to WM. This concept was recently extended by a large study that involved 1,539 patients with WM and 605 patients with LPL. It showed that first-degree relatives of patients with LPL/WM had a significantly increased risk of developing (1) WM themselves, (2) MGUS (including IgG^+^ and IgA^+^), or (3) a type of NHL that is related to WM in terms of biology and clinical presentation (e.g., B-CLL) [84]. The result supports the existence of common B-lineage cancer susceptibility alleles that render carriers susceptible to both LPL/WM and a number of “kindred” lymphoproliferative disorders [85, 86]. A dominant or codominant genetic trait appears to be more likely for these alleles than a recessive trait [87]. An important but difficult task is the identification of common lymphoma and WM alleles. Projects of this nature often begin with microarray-based genome-wide linkage analysis a.k.a. GWAS (genome-wide association studies) of affected individuals. The first analysis was successfully completed by Mary McMaster and her associates. They investigated 11 families at high risk for WM. Included were 122 individuals, of which 34 and 10 had developed frank WM and IgM MGUS, respectively. The strongest evidence of linkage was found on chromosomes 1q and 4q, but additional WM loci are suspected on chromosomes 3 and 6 [88]. This approach may also be helpful to identify genetic markers that predict progression from IgM MGUS to frank WM [89], a critical knowledge gap. Another priority for future research is the elucidation of gene and environment interactions; that is, how do genetic polymorphisms in the germ line (WM alleles) cooperate with environmental factors (e.g., infectious agents) to drive WM? Well designed and sufficiently powered population-based studies [90] may be key for further progress on the elucidation of genetic networks that predispose to WM. 

### 2.4. Tumor Genetics and Microenvironment

From a biochemical perspective, WM cells exhibit heightened and/or constitutive activation of cellular signal transduction pathways that have been implicated not only in the growth, proliferation, and survival of malignant B cells, but also in the acquisition of resistance to therapeutic agents in patients with WM. Signaling pathways of this sort include PI3 K-AKT-mTOR [91], NF*κ*B [92, 93], Src tyrosine kinases [94], BCL6-BLIMP1-XBP1 [95], and SDF-1/CXCR4/VLA-4 [96]. The biological factors that drive elevated signaling activity in WM are poorly understood, but some of the genetic factors have been revealed in genomic studies of WM. These include *PRDM1*, which encodes the master regulator of B cell and plasma cell differentiation, BLIMP1; *TNFAIP3* (tumor necrosis factor, alpha-induced protein 3), which is better known as A20; and, rarely, *TRAF3*, which encodes the adapter protein, TNF receptor-associated factor 3 [97]. Exciting new lines of investigation concern the deregulated expression of micro-RNAs (miR) in WM cells, with miR-155 receiving the greatest attention at this time [98], and the highly recurrent mutation in the MYD88 adapter protein that replaces a leucine (L) residue at position 265 with a proline (P) [99]. The significance of MYD88^L265P^ will be discussed in greater depth in Section 5.

Widely recognized in the WM community is the crucial role the bone marrow microenvironment plays in tumor development and maintenance [100]. Bone marrow from WM patients contains elevated numbers of mast cells [101], which use CD70 (a ligand for the TNF-receptor superfamily member, CD27) on the cell surface to bind soluble CD27 (sCD27) secreted by tumor cells [102]. The CD27-CD70 interaction activates mast cells to produce TNF family proteins, such as CD40L, APRIL, and BLYS, which, in turn, stimulate WM cells to release more CD27, thereby establishing a classic feed-forward cytokine activation loop between mast cells and tumor cells. There is widespread agreement on the importance of the proinflammatory cytokine, IL-6, for WM. IL-6 is mainly produced by bone marrow stromal cells (BMSCs) in a paracrine fashion. It binds to the IL-6 receptor (composed of gp80 and gp130) on tumor cells where it activates Jak-Stat3 signaling. This leads to secretion of CCL5 (chemokine C-C motif ligand 5 a.k.a. RANTES), which binds to CCR3 (C-C chemokine receptor type 3 or CD193, a member of family 1 of the G protein-coupled receptors) on BMSCs where it stimulates more IL-6 production. The IL-6/CCL5 axis is thus another feed-forward cytokine loop between tumor and bystander cells in WM. IL-6 is not only upregulated in tumor cells [103] but—together with its target, C-reactive protein (CRP)—present at elevated levels in the sera of WM patients [104, 105]. WM cells also interact with bone marrow endothelial cells (BMECs) by virtue of Ephrin-B2/Eph-B2 signaling [100], a pathway for increased adhesion of tumor cells to endothelial cells and, perhaps, for cell adhesion-mediated drug resistance in patients undergoing chemotherapy. Homing of WM cells to the bone marrow is regulated, in part, by the BMSC-secreted chemokine SDF-1 (stromal cell-derived factor 1 that is officially designated chemokine [C-X-C motif] ligand 12 or CXCL12), which binds to the CXCR4 receptor (CD184, fusin) on the tumor cells. Last but not least, WM cells also interact with bone marrow extracellular matrix components, triggering signaling pathways in tumor cells that are less well known but deserving of additional study [100]. 

### 2.5. Key Points and Future Directions

**Figure 2:**
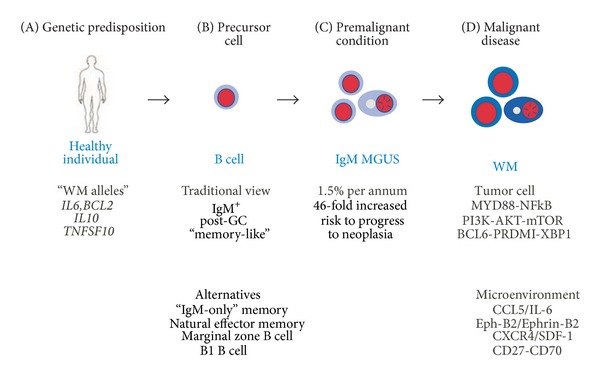
Natural history of WM. (A) One major gap in our current understanding of WM is that no definitive cause has been identified. Although autoimmune and inflammatory conditions have been shown to increase the risk of WM [107], the fundamental biological mechanism underlying risk elevation by these conditions remains unclear. Early observations on familial clustering of WM have prompted additional studies on hereditary predisposition in affected families, case-control studies, and cohort studies—all with the goal of identifying the underlying “WM genes” or—to use a more appropriate scientific term—the susceptibility alleles of WM [108]. This effort has uncovered allelic variants of *IL10*, *TNFSF10*, *IL6*, and *BCL2* as candidate WM genes [109]. The latter two are of particular interest because an increase in *BCL2* expression has been implicated in enhanced B-lymphocyte survival and hypergammaglobulinemia in familial WM [110], and IL-6 is upregulated in LPL-WM cells [111]. (B) The traditional view postulates that WM is derived from an antigen-experienced, post-GC “memory-like” B lymphocyte, but more recent insights into IgM memory have raised doubt whether GC passage is a requirement for generating WM precursors. Like normal IgM^+^ memory B cells, which can mature to IgM-secreting plasma cells in the bone marrow [112], WM cells differentiate into lymphoplasmacytic cells and plasma cells in the bone marrow [113]. Clonally related IgM^+^ B cells are also detected in the peripheral blood of WM patients, and their numbers increase in patients who progress or fail to respond to therapy [114]. In IgM MGUS patients, these cells possess the peculiar capacity to differentiate spontaneously, in an IL-6-dependent manner, into plasma cells [115]. In WM patients, however, this differentiation is largely independent of IL-6 [115]. Elucidating the mechanism responsible for the switch from IL-6 dependence to independence is an outstanding question in WM research. (C) IgM MGUS is a premalignant expansion of a single clone of aberrant lymphoplasmacytic cells that often precedes the emergence of frank WM [116]. IgM MGUS confers, on average, a 46-fold elevation of the risk to progress to WM [74]. Evidence for a genetic predisposition to MGUS is currently emerging [115, 117, 118]. Identification of the genes or pathways that drive the transition from precursor B cell to IgM MGUS and the subsequent progression of IgM MGUS to WM may lead to new interventions in WM. (D) Global gene expression profiling (GEP), array-based comparative genomic hybridization (aCGH), fluorescence *in situ* hybridization (FISH), and other genomic and cytogenetic methods have advanced our understanding of the changes in the LPL-WM genome [119]. For example, cytogenetic studies uncovered recurrent deletions of chromosome 6q, an adverse prognosticator for WM patients, presumably due to the loss of an important yet unidentified WM suppressor gene [120]. GEP first suggested that WM is very similar to CLL [111]; however, as pointed out in a commentary [116], further analysis of bone marrow cells that were first fractionated into CD19^+^ and CD138^+^ subpopulations by fluorescence-activated cell sorting (FACS) [103] revealed that this similarity is only superficial. Array CGH revealed that deletion of TRAF3 (a possible cause of NF*κ*B activation in WM) and a loss of miR15/16 (two microRNAs) are involved in BCL-2 upregulation in tumor cells [121]. MicroRNA profiling [122, 123], antibody-based protein arrays [124], and other emerging methods will no doubt reveal additional changes in the WM genome. In analogy to many other types of cancer, the new results will likely lead to the identification of genetic subgroups of LPL-WM and to patient stratification based thereon.

A simplified scheme on the pathophysiology of WM is depicted in Figure 2. Despite encouraging progress in the past decade, our knowledge of the natural history of IgM^+^ LPL remains superficial. This is particularly true of the etiology of and genetic predisposition to WM, as well as the sequence of genetic and epigenetic changes that underlie malignant transformation of WM cells. Thanks to the availability of a sophisticated armamentarium of molecular cytogenetic methods (e.g., fluorescence in situ hybridization [FISH] and spectral karyotyping [SKY]) and high-throughput genomic methods—such as array-based global gene expression profiling, single-nucleotide polymorphism (SNP) analysis, comparative genomic hybridization on microarrays (aCGH) and, more recently, whole-exome and whole-genome sequencing—we have an increasingly detailed understanding of the WM oncogenome. Our appreciation of the tumor microenvironment has also increased, although many pieces of that puzzle are still missing. Excellent minireviews on the subject matter covered in this chapter are available in a 2013 proceedings issue of *Clinical Lymphoma*,* Myeloma* & *Leukemia* published on the occasion of the 7th International Workshop on WM held in Newport, Rhode Island [106].

## 3. Immunophenotype and Immunological Specificity

### 3.1. Immunophenotype

WM cells exhibit an immunophenotype that is consistent with a mature IgM^+^ B cell that is poised to enter into the pathway of plasmacytic differentiation, but for reasons that are not obvious, is unable to complete it. The *κ* to *λ* light-chain usage of the hallmark mIgM protein is skewed in favor of the former, with a ratio of 5 to 1. In serum of healthy adults, this ratio is 2 to 1. The reason for the biased utilization of *κ* light chains is not known.

The lymphocytoid portion of the tumor cell clone typically expresses surface IgM in conjunction with pan B cell markers, including CD19 (a B cell receptor [BCR] coreceptor that decreases the threshold for antigen-dependent stimulation of BCR signaling), CD20 (a phosphoprotein that optimizes humoral immune responses to T-independent [TI] antigens), CD22 (a sialic acid-binding, immunoregulatory transmembrane lectin that prevents overactivation of the immune system and development of autoimmunity), and CD79a (an accessory component of the BCR known as Ig*α*). Nuclear immunoreactivity of lymphocytoid cells includes BCL2 (B cell CLL/lymphoma 2, an antiapoptotic oncoprotein) and PAX5 (paired box 5 a.k.a. B cell specific activator protein, which is important for B cell development and maintenance of lineage fidelity). The cluster of differentiation (CD) antigen CD5 (hallmark of B1 B cells that mitigates activating signals from the BCR) is variably expressed; a recent study reported 5% to 20% of cases to be positive (usually weakly) for this surface marker. CD10, a membrane metalloendopeptidase that is also known as neprilysin or common acute lymphoblastic leukemia antigen (CALLA), as well as CD23 and CD103 (*ITGAE*-encoded integrin) are mostly absent. The plasmacytoid portion of the tumor cell clone typically expresses cytoplasmic IgM that is secreted into the serum, in conjunction with B cell markers; for example, CD19 and CD45 (protein tyrosine phosphatase, receptor type, C or PTPRC) and plasma cell markers; for example, CD38 (cyclic ADP ribose hydrolase) and CD138 (syndecan 1). This indicates that the cells are arrested at an intermediate plasmablast-like or preplasmacyte-like stage of differentiation. Nuclear immunoreactivity includes IRF4 (interferon regulatory factor 4) and PRDM1 (PR domain containing 1, with ZNF domain, better known as BLIMP1). 

In agreement with the flow cytometric pattern described previously, a recent immunohistochemical study of nuclear protein expression of WM cells demonstrated that less mature CD138^+^PAX5^+^ plasma cells were significantly more abundant in WM than in marginal zone lymphoma (MZL) or plasma cell myeloma (a.k.a. multiple myeloma or MM). Conversely, more mature CD138^+^IRF4^+^ cells were rare in WM relative to MZL and myeloma [125]. The transcription factor network that governs the peculiar lymphoplasmacytic differentiation arrest of WM cells is poorly understood.

### 3.2. Immunoglobulin Analysis

DNA sequence analysis of productively rearranged, expressed Ig variable (IgV) genes has provided important clues on the natural history of CLL, SMZL, MCL, and other neoplasms derived from mature B lymphocytes [126–129]. Inspired by these advances, investigators analyzed the variable portion of IgM heavy- and light-chain genes from both patients with WM and individuals with IgM MGUS. In addition to the skewed *κ*/*λ* light-chain ratio mentioned previously, research questions included the mutational status of IgV genes (germ line versus mutated versus hypermutated), the immune repertoire utilized by WM cell clones (random versus biased usage of V_H_ or V_L_ gene families and/or individual genes within these families), the ability of WM cells to perform class switch recombination (CSR; i.e., switching of heavy-chain constant region genes from the “unswitched” or “preswitch” *μ*/*δ* isotypes to the “switched” or “postswitch” *γ*, *α*, and *ε* isotypes), and the propensity of WM to undergo clonal diversification (intraclonal heterogeneity). The results of these investigations showed that the great majority of mIgM IgV genes harbor somatic mutations, although the number of mutations was low in many cases. Additionally, there was evidence for nonrandom IgV gene usage; for example, genes from the V_H_3 family were more frequently involved in WM than one would expect by chance. There was little if any support for ongoing diversification of WM cell clones; in other words, the neoplasm appears to be remarkably stable with regard to Ig expression [130–134]. Although WM cells could be coaxed by some investigators to undergo CSR *in vitro*, this appeared to take place at a very low level [135] and, with the exception of one well-documented example [136], did not seem to occur *in vivo*. A recent examination of 59 patients with WM and 64 individuals with IgM MGUS (*n* = 123) reconfirmed the older studies summarized previously. The productively rearranged VDJ_H_ region of IgM was molecularly cloned in 99 of 123 (80%) cases and then analyzed using DNA sequencing: V_H_ genes were mutated in 94 of 99 (95%) cases. The median rate of mutation was 6.7% (6.7 base-substitution changes per 100 nucleotides sequenced), with a range from 2.1% to 14.5%. Compared to normal B cells, genes from subgroup V_H_3 were overrepresented in both WM and IgM MGUS cells, whereas genes from subgroups V_H_1 and V_H_4 were underrepresented. The results were interpreted to mean that WM cells have antigen experience [137]. The nature of the underlying antigens will be discussed in the following two sections.

### 3.3. Autoimmune and Natural Antibodies

Determination of the immunological specificity of the hallmark monoclonal IgM protein may lead to additional insights into the natural history of WM, because it may reveal the underlying antigens that stimulated WM precursors by virtue of BCR signaling in the course of tumor development. As alluded to in Section 1.3, the clinical presentation of patients with WM or individuals with IgM MGUS sometimes provides clues about the immunological specificity of the associated IgM [138]. This is the case when the immunoglobulin is autoreactive and, therefore, functions as an antibody that can bind to a specific self-antigen and cause autoimmunity. WM has been firmly linked to three autoantibody syndromes designated mixed IgM-IgG cryoglobulinemia (MC) [139], chronic cold agglutinin disease (CAD) [140], and IgM neuropathy [141]. The associated self-antigens include, respectively, the Fc portion of IgG, so-called I/i epitopes on red cells, and neural carbohydrates. However, it is doubtful whether these autoantigens represent the original ligands that induced IgM signaling in WM precursors and, thereby, promoted oncogenesis. Instead, evidence indicates that the original antigens are exogenous in nature, possibly including hepatitis C virus in case of MC, bacterial LPS in case of CAD, and various bacteria and viruses in the neuropathies [138]. This would be in line with our current understanding of the etiology of WM presented in Section 2.2. The literature on WM paraproteins includes numerous case reports on additional mIgM autoantibody specificities, suggesting that the three autoimmune syndromes mentioned previously comprise only the tip of the iceberg. Examples of autoantibodies include reactivity to cytomegalovirus [142] and, as described decades ago by Jan Waldenström himself, to streptolysin-O [143]. This provides additional ammunition for implicating exogenous antigens in WM.  Section 4.4 will discuss the possibility that WM-associated autoantibodies, and perhaps many WM-associated IgM paraproteins for which the immunological specificity remains unknown, are in fact natural antibodies [144] produced by natural effector memory B cells. Natural antibodies, which can be readily detected in neonates and young children without prior exposure to pathogen, are immunoglobulins that exhibit low affinity to antigen and polyreactivity to a large number of self and non-self antigens. In conclusion, autoimmune symptoms of patients with WM may be brought about by cross-reactivity of IgM to exogenous and endogenous antigens.

### 3.4. T-Cell-Selected mIgM

Investigators in Germany have recently come up with an alternative explanation for the origin of WM-associated paraproteins; they proposed that the mIgM is in fact a product of a T-cell-dependent (TD) immune response. They used a relatively new method of Ig analysis called expression cloning [145] to determine the immunological specificity of IgM paraproteins from patients with WM and individuals with IgM MGUS and discovered several common self-antigens dubbed “paraprotein targets” or “paratargs” for short, including a phosphorylated form of paratarg-7, which they termed pP-7. Compared to noncarriers, carriers of pP-7 were found to have a 6.5-fold higher risk for developing IgM MGUS and WM [146], as well as an 8-fold to 13-fold elevation of the risk for IgA/G MGUS and MM [147]. Biochemical studies showed that phosphorylation of P-7 occurred at serine 17 (S17), a residue that is strategically located in the paraprotein-binding epitope of the self-antigen. Analysis of the CD4 T-cell response to both P-7-(S17 not phosphorylated-) derived peptides and pP-7-(S17 phosphorylated-) derived peptides in affected and nonaffected family members demonstrated that pP-7 induced a strong HLA-DQ- and HLA-DR-restricted immune response, but P-7 did not. This suggested that pP-7 confers sustained autostimulation of cognate helper T cells, which, in turn, specifically activate B cells with high-affinity binding to pP-7. These B cells appear to undergo clonal expansion until IgM MGUS is manifest and progresses, in some cases, to frank WM. Preliminary coimmunoprecipitation studies presented at ASH 2012 identified PKC*ζ* (protein kinase zeta) and PP2A (protein phosphatase 2A) as the principal kinase and phosphatase at P-7 S17, respectively. These results indicate that only those pP-7-carrying individuals are at elevated risk of developing WM that have the requisite MHC (major histocompatibility complex) class II haplotype for cognate interaction of antigenic pP-7 peptides and CD4^+^ T cells. The investigators speculate that this may explain, in part, the different prevalence of IgM MGUS/WM in different ethnic backgrounds. The findings presented previously raise the possibility that WM precursors are generated in TD immune responses.

### 3.5. Key Points and Future Directions

**Figure 3:**
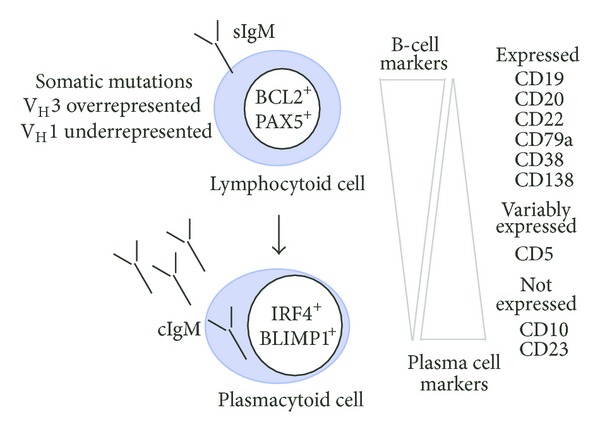
Immunophenotype of WM. See Section 3 for details.

The immunophenotype of WM is well established (Figure 3), and the autoantibody activity of WM-associated paraproteins is commonly known. Still lacking, however, is a detailed analysis of the immunological specificity of IgM paraproteins with respect to primordial, underlying antigens. This is a difficult area of research, plagued with a host of technical problems that has not been productive in the past. This will hopefully change in the near future as—in addition to expression cloning [145] used in the “paratarg” studies described in Section 3.4—new methods have been developed that lend themselves to identifying and validating specific antigens/haptens recognized by WM-associated mIgM. Epitope-mediated antigen prediction (E-MAP) [148] and high-density peptide microarrays [149] are two promising methods to that end. It is possible that both endogenous (self) antigens and exogenous (microbial) antigens trigger WM. Elucidating the nature of these antigens is of great relevance for the long-standing, thorny issue of the cellular origin of WM. This will be discussed in depth in the following chapter.

## 4. Cell of Origin

### 4.1. WM Precursors May Derive from the IgM^+^ Memory B-cell Compartment

The immunophenotypic profile of WM cells—in conjunction with the occurrence of somatic mutations in *IGV*-encoded portions of the IgM paraprotein heavy and light chains—has long been interpreted to mean that WM originates from a mature, antigen-experienced B cell that exits the GC as an atypical IgM^+^ memory B cell that had engaged the somatic hypermutation (SHM) pathway of antibody affinity maturation but failed to perform CSR in the course of the GC reaction [131]. This view, which implies that the WM precursor is a conventional follicular B cell recruited into a TD immune response, has been challenged by recent progress in our understanding of the IgM^+^ memory B cell compartment, which appears to be more complex than assumed before. Indeed, new pathways of IgM^+^ B cell differentiation and memory formation discovered in the past few years [150, 151] prominently include so-called unconventional B cells, such as MZ B cells and B1 B cells, in addition to conventional, follicular B cells often referred to as B2 cells. The newly appreciated complexity of IgM^+^ memory has raised the bar higher for identifying WM precursors, because it is possible that the precursor cell pool is heterogeneous and gives rise to different subtypes of WM depending on the underlying B cell.

**Figure 4:**
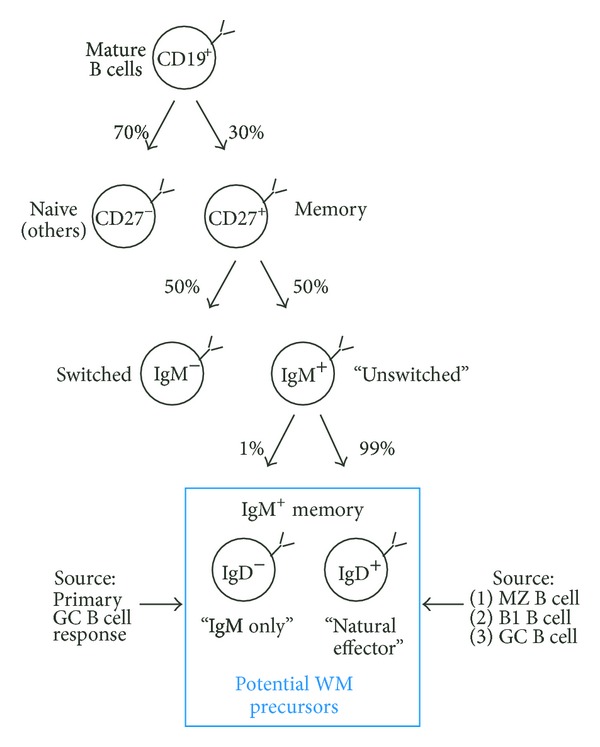
Possible origin of WM precursors from IgM^+^ memory cells. Of human splenic and peripheral blood B cells, ~70% are CD27^−^ and ~30% are CD27^+^. The CD27^−^ cells comprise a mixed population that contains both naive IgM^+^ B cells that express germ line IgV genes and IgG^+^/IgA^+^ memory B cells that harbor mutated IgV genes (not shown). Naive B cells have not yet participated in an immune response and not yet modified their expressed IgV genes using the somatic hypermutation (SHM) pathway. In contrast, memory B cells are immunologically experienced and have engaged the SHM machinery to increase the affinity of the expressed IgV gene to the underlying antigen. The CD27^+^ compartment is composed equally of “switched” IgM^−^ B cells and “unswitched” IgM^+^IgD^+^ B cells. The former have performed H chain class switch recombination (CSR) and, thereby, replaced their *μ*H with *γ*/*α*/*ε*H chains, while the latter have not. The great majority of CD27^+^IgM^+^ cells coexpress IgD (IgM^+^IgD^+^)—a compartment that is often referred to as “natural effector” memory. CD27^+^IgM^+^IgD^−^ B cells, sometimes called “IgM-only” cells for short, only represent a minor population (1%) in peripheral blood and spleen. Both CD27^+^IgM^+^IgD^+^ and CD27^+^IgM^+^IgD^−^ memory B cells may be precursors of WM, although the weight of the evidence seems to favor the IgM^+^IgD^+^ compartment as the stronger candidate. This compartment likely constitutes a mixture of marginal zone (MZ)-, B1- and germinal-center- (GC-) derived B cells. The CD27^+^IgM^+^IgD^−^ compartment is better defined as it originates from a single source: the primary GC response, which often selects for mutated *IGKV3-20* alleles.

The sources of IgM^+^ memory B cells and their abundance in spleen and peripheral blood are depicted in Figure 4. It shows that flow cytometric analysis of mature CD19^+^ B-lymphocytes for expression of the memory B cell marker, CD27, identifies approximately 30% of cells as CD27^+^ memory B cells [152]. CD27 is a member of the TNF receptor superfamily that binds to CD70, a member of the TNF ligand superfamily [152, 153]. The CD27^+^ compartment is composed equally of “switched” IgM^−^ B cells and “unswitched” IgM^+^ B cells that have or have not undergone CSR, respectively. The CD27^+^IgM^+^ compartment can be further divided based on expression of surface IgD, an immunoglobulin that is expressed at low levels when B lymphocytes exit the bone marrow and begin to populate peripheral lymphoid tissues but is upregulated and strongly expressed when the cells reach full maturity* in situ*. Double-positive IgM^+^IgD^+^ or “natural effector” cells comprise the bulk of the CD27^+^IgM^+^ compartment (99%), whereas single-positive IgM^+^IgD^−^ or “IgM-only” B cells only represent a minor population (1%) [150]. IgM^+^IgD^−^ B cells are thought to be products of primary GC responses involving follicular B cells, whereas IgM^+^IgD^+^ B cells likely constitute a mixture of unconventional and conventional B cells [154]. 

It seems reasonable to propose that the IgM^+^ memory compartment (indicated by blue box in Figure 4) contains the elusive WM precursor(s). But even if that was true, the question remains whether WM is derived from an IgM^+^IgD^−^ B cell or IgM^+^IgD^+^ B cell. While the former nicely matches the IgM^+^IgD^−^ phenotype of a typical WM cell, it is rare to begin with. The latter does not match the WM surface marker profile but is abundant and easily reconciled as a WM precursor if one postulates that IgD is downregulated or lost during malignant cell transformation. Consistent with this possibility, a minority of cases (5–10%) weakly expresses IgD (Figure 2), and CD20^+^IgM^+^IgD^+^ B cells have been implicated as WM precursors in the past [155]. The biological significance of the IgM^+^ memory B cell compartment and its principal developmental pathways will be discussed in the following in greater depth.

### 4.2. B cell Memory Is Long Lived and Includes a Sizeable Fraction of IgM^+^ Cells

Human memory B cells can survive for decades [156–158]. This is intriguing from a tumor development point of view because longevity can be expected to endow a putative target of neoplastic development with greater opportunities to complete the time-consuming, multistep process of oncogenesis than available for short-lived B cells. The life span of the latter may be insufficient to accumulate all the genetic and epigenetic changes required for fully autonomous, malignant growth. Upon reencounter with the antigen or hapten for which they exhibit immunologic specificity, memory B cells can rapidly proliferate and differentiate into plasma cells that secrete high-affinity antibodies. Memory B cells have classically been defined as antigen-experienced, long-lived IgD^−^ B cells that have undergone somatic mutations in their productively rearranged and expressed *IgV *genes. Additional characteristics include the presentation, on the cell surface, of the memory marker, CD27, and costimulatory and activation markers, including CD80 and CD86 (tandem ligands for CD28 and CD152 on T cells, resp.), as well as CD95 (FAS receptor). In contrast to these markers, the low-affinity IgE receptor, CD23, is downregulated in memory B cells [159, 160]. 

Recent work from several laboratories has demonstrated that the memory B cell population is phenotypically, functionally, and ontogenetically heterogeneous. Analysis of Ig expression patterns by antigen-binding CD27^+^ B cells revealed that a surprisingly large proportion of memory cells do not perform CSR of the expressed H chain and, therefore, continue to express IgM. This finding not only demonstrated the existence of IgM^+^ memory [161, 162], it also raised the question what the benefit for the host might be to maintain these cells for long periods of time. One important answer came from a study in mice, showing that upon secondary challenge with antigen, the IgM and IgG memory subsets displayed differential effector functions: the IgG^+^ cells rapidly differentiated into plasmablasts, whereas the IgM^+^ cells reinitiated secondary GC reactions [163]. This suggested that IgM^+^ cells comprise a durable reserve for humoral immune responses that can be recalled at an indeterminate, later point in life. A related, independent study demonstrated that IgM^+^ cells were longer lived than their IgG^+^ counterparts but responded to reencounter with the specific antigen in a different way: the switched memory dominated the secondary immune response (due to its capacity to become activated in the presence of neutralizing serum Ig), whereas IgM^+^ memory cells did not contribute to this response until after the switched memory cells disappeared and the Ig levels had declined [164]. This result led to the theory that IgM^+^ memory is “waiting in the wings,” as a backup force of sorts, ready to spring into action when the switched memory fails to clear the antigen but quietly staying in the background when the switched memory is able to take care of the job [164].

### 4.3. “IgM-only” or IgM^+^IgD^−^ Memory B cells

Features, subtypes, and developmental history of IgM^+^ memory B cells have been worked out in detail in the past few years (Figure 4, bottom). B cell immunologists now divide IgM^+^ memory B cells into two compartments: “natural effector” cells, which will be described in Section 4.4, and “IgM-only” cells, which will be described here. Evidence provided by a Dutch group indicates that “IgM-only” or CD27^+^IgM^+^IgD^−^ memory B cells originate from primary GC responses. Building on the widely accepted paradigm that extensive antigen-induced cell proliferation and somatic hypermutation (SHM) are hallmarks of memory B cells, the Dutch investigators developed two nifty molecular assays—called KREC (Ig*κ*-deleting recombination excision circle) and Ig*κ*REHMA (Ig*κ* restriction enzyme hot-spot mutation)—that are able to generate good estimates of a B cell's replication history (i.e., number of cell divisions) and SHM history (i.e., number of somatic mutations in IgV genes), respectively [165]. KREC is a qPCR assay for determining the ratio of genomic coding and signal joints formed in consequence of genetic inactivation of the *IGK* locus, which is accomplished by chromosomal rearrangements that delete the *IGK* constant region gene, C*κ*, together with the downstream *IGK* enhancer, 3′E*κ* [165]. Ig*κ*REHMA is a DNA spectratyping assay that determines the mutational load in CDR1 (complementarity determining region 1) of the *IGKV3-20* gene, which has been validated as a reliable indicator of overall SHM activity in expressed Ig genes *in vivo*. The Dutch investigators found that “IgM-only” B cells had a replication and SHM history in line with GC B cells, suggesting that CD27^+^IgM^+^IgD^−^ B cells are offspring of follicular B cells that underwent SHM-mediated affinity increases in their *μ*H chains in the course of a primary GC response but exited the GC prior to undergoing isotype switching. “IgM-only” memory B cells match the IgM^+^IgD^−^ phenotype of frank WM and have been repeatedly shown to undergo expansion in patients with autoimmune diseases [166, 167]—a putative driver of WM. This backdrop suggests that the natural history of IgM MGUS and WM may begin with “IgM-only” memory B cells.

### 4.4. “Natural Effector” or IgM^+^IgD^+^ Memory B cells

CD27^+^IgM^+^ memory B cells that coexpress IgD outnumber their IgD^−^ counterparts in peripheral blood and spleen by approximately two orders of magnitude. CD27^+^IgM^+^IgD^+^ or “natural effector” B cells comprise a heterogeneous compartment that includes developmentally primitive, unconventional B cells derived from MZ B cells or B1 B cells [150, 151]. The “primitive” origin notwithstanding, natural effector B cells are thought to be *bona fide* memory cells based on three lines of evidence. First, they express high levels of activation/costimulation molecules such as CD80, CD180 (also known by the archaic term Bgp-95 [168]), and TACI (tumor necrosis factor [TNF] receptor superfamily, member 13B [TNFRSF13B]). CD180 and TACI are of special interest because they induce cellular signal transduction pathways dependent on myeloid differentiation primary response 88 (MYD88). CD180, which associates with lymphocyte antigen 86 (LY86 a.k.a. MD-1) to form a cell surface receptor complex that belongs to the Toll-like receptor (TLR) family, works in concert with TLR4 to regulate B cell activation in response to lipopolysaccharide (LPS), a membrane constituent of Gram-negative bacteria. TACI regulates humoral immunity by virtue of interacting with two TNF family ligands, BAFF (B cell activating factor) and APRIL (a proliferation-inducing ligand). BAFF [169]—also known as TNF ligand superfamily, member 13B (TNFSF13B), B-lymphocyte stimulator (BLYS), TNF- and APOL-related leukocyte expressed ligand (TALL1) [170], or cluster of differentiation 257 (CD257)—is a cytokine that acts as a potent activator of B cells and plays an important role in the proliferation and differentiation of B cells. APRIL—also referred to as tumor necrosis factor ligand superfamily, member 13 (TNFSF13) or cluster of differentiation 256 (CD256)—is important for the long-term survival of plasma cells in the bone marrow [171]. The BAFF/APRIL/TACI/MYD88 axis is important for the development of natural effector cells and their function as memory cells. Second, natural effector B cells appear to be selected against inherently autoreactive V_H_ domains, a general feature of memory B cells. Consistent with that, the cells have an extensive replication history compared to naive B cells (using the KREC assay) and exhibit SHM profiles of Ig heavy and light variable genes that are characterized by high R/S (replacement to substitution) ratios in H chain CDRs (Ig*κ*REHMA assay) [154]. Natural effector B cells were found to have proliferated somewhat less vigorously (7 cell divisions on average) than GC B cells (9 divisions), but the mutational load in *IGHV *was comparable in the two cell types. This result lent additional support to the contention that a substantial fraction of CD27^+^IgM^+^IgD^+^ memory B cells is generated outside the GC, using a pathway of SHM that operates independently of the GC reaction. Third, a recently reported high-throughput analysis of V_H_ sequences from blood samples of three adult donors [172] found major differences in the immunological repertoire of CD27^+^IgM^+^IgD^+^ B cells and IgG^+^ or IgA^+^ B cells. Compared to switched B cells, natural effector B cells exhibited a twofold higher usage of V_H_3 but a 20-fold lower usage of V_H_1. The marked bias for (V_H_3) and against (V_H_1) heavy-chain gene families is strikingly similar to that seen in WM, raising the possibility that the same selective force might shape the repertoire of natural effector and WM cells [172]. These findings lend credence to the proposal that WM is derived from natural effector B-memory cells.

### 4.5. Key Points and Future Directions

The IgM^+^ memory B cell compartment may contain the elusive WM precursor. IgM memory consists of a minor fraction of “IgM-only” cells and a predominant fraction of “natural effector” B cells. Both are viable candidates for giving rise to WM.

## 5. MYD88 Signaling Links Natural Effector Memory and WM

### 5.1. Genetic Defects in the MYD88-IRAK4-TIRAP Pathway Result in Loss of IgM^+^IgD^+^ Memory B cells

**Figure 5:**
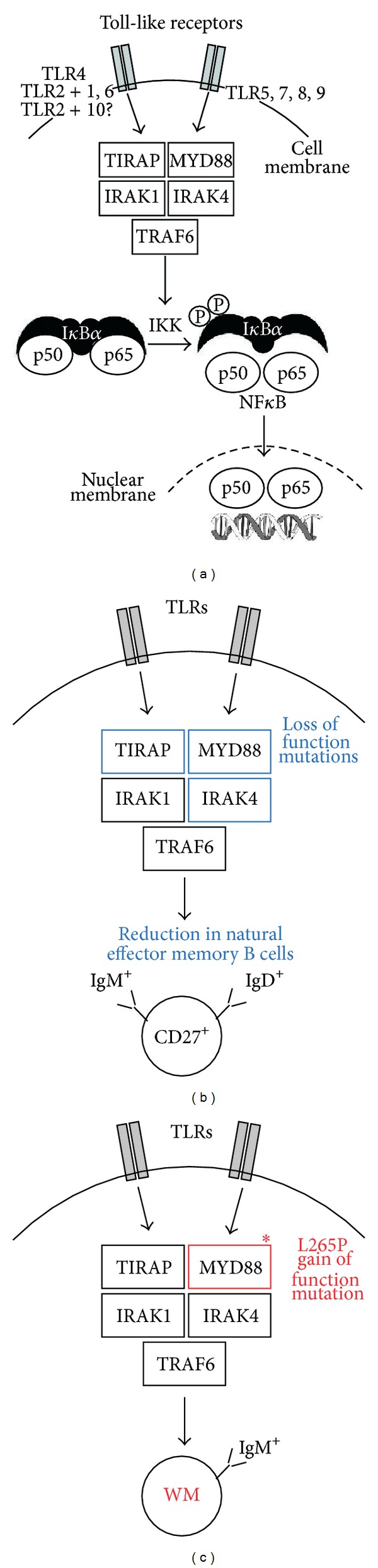
MYD88—an intriguing link between WM and natural effector IgM^+^ memory cells. (a) In normal B-lymphocytes and other cells of the immune system, MYD88 functions as a signaling adaptor protein that activates the nuclear factor *κ*B (NF-*κ*B) pathway following stimulation of Toll-like receptors (TLRs) and receptors for IL-1 and IL-18 (not shown). MYD88 coordinates the assembly of a supramolecular signaling complex that contains members of the IRAK (interleukin-1 receptor-associated kinase) family of serine-threonine kinases. Following TLR ligand binding, MYD88 is recruited to the cell membrane-bound receptor complex, leading to recruitment of IRAK4, which activates IRAK1 and IRAK2 (not shown) by phosphorylation of serine and threonine residues [177, 178]. IRAK1-dependent activation of tumor necrosis factor receptor-associated factor 6 (TRAF6) affects NF-*κ*B activation via phosphorylation of I*κ*B*α* and execution of the NF-*κ*B-dependent gene regulation program. TIRAP is an additional adapter protein involved in TLR4 and TLR2 signaling (see Section 5.1 for details). (b) Genetic loss of MYD88, IRAK4, and TIRAP function leads to significant reductions in CD27^+^IgM^+^IgD^+^ memory B cells, indicating that the MYD88-TIRAP-IRAK4 pathway is essential for homeostatic maintenance of the natural effector memory. (c) WM cells harbor the highly recurrent, gain-of-function L265P exchange in the MYD88 adaptor protein, indicating that WM depends on elevated TLR signaling. This is intriguing in light of the findings depicted in (b) and suggests that WM is derived from natural effector memory B cells.

Inherited immunodeficiency disorders may be crudely considered experiments of Mother Nature that permit us to glean unique insights into pathways of immunity that would be difficult to dissect in immunocompetent individuals. This is particularly true for IgM memory, the understanding of which has been greatly aided by the examination of patients that harbor rare genetic defects in immune genes governing memory responses. An important early clue was obtained in investigations of patients with deficiencies in GC formation due to lack of CD40-CD154 signaling during cognate B- and T-cell interaction at the outset of the GC reaction. The number of CD27^+^IgM^+^IgD^+^ memory B cells was significantly reduced in these patients, yet the cells did not completely disappear from peripheral blood [173–175]. This finding indicated that CD27^+^IgM^+^IgD^+^ memory B cells are normally generated using both GC-dependent and -independent pathways. Postulating that the latter include pattern recognition receptors of the Toll-like receptor (TLR) family, a recent study analyzed peripheral B cell subsets in patients exhibiting genetic defects in TLR signaling. Inherited deficiencies along this line affected (1) one of ten TLRs expressed in human beings: TLR3, (2) three different adapter proteins: MYD88, toll-interleukin 1 receptor domain containing adaptor protein (TIRAP), and toll-like receptor adaptor molecule 1 (TICAM a.k.a. C. elegans unc-93 homolog B1 or UNC-93B), and (3) a protein kinase involved in downstream signal transduction: interleukin-1 receptor-associated kinase 4 (IRAK4). In order to put the biological consequences of these genetic defects into perspective, it is important to realize that, first, all TLRs except TLR3 rely on MYD88 and IRAK4 for downstream signaling and, second, TIRAP is additionally important for signaling induced by TLR4 homodimers and the heterodimers TLR2 forms with TLR1, TLR6 and, possibly, TLR10. This is schematically depicted in Figure 5(a). The analysis revealed that loss of MYD88, IRAK4, and TIRAP function led to significant reductions in CD27^+^IgM^+^IgD^+^ memory B cells, but left their IgD^−^ counterparts unchanged (Figure 5(b)). The discovery of MYD88-TIRAP-IRAK4 as the first genetic pathway specifically required for CD27^+^IgM^+^IgD^+^ memory [176] lends support to the concept that pattern recognition receptors signaling through MYD88 homodimers (Figure 5(a), right) or MYD88/TIRAP heterodimers (Figure 5(a), left) promote the homeostatic maintenance of B cells that do not depend on T-cell help for activation and survival. Sections 5.2 and 5.3 will argue that the decrease in circulating MYD88-TIRAP-IRAK4-dependent CD27^+^IgM^+^IgD^+^ memory B cells shown in Figure 5(b) may be the result of impaired survival and immunological responsiveness of MZ and B1 cells [154, 176]—even though alternative explanations (Section 5.4) exist.

### 5.2. IgM^+^IgD^+^ Memory B cells May Be Derived from MZ B cells

MZ B cells are good candidates for MYD88-TIRAP-IRAK4-dependent natural effector memory B cells for the following reasons. First, unlike laboratory mice in which MZ B cells express germline *IGV* genes, reside in the splenic marginal zone, and do not recirculate [166], MZ B cells in human beings carry mutations in the *IGV*-encoded portions of the B cell receptor and recirculate in the blood stream [166]. The mutations in *IGV* genes appear to be of the more limited “extrafollicular variety” [154, 166] and are probably caused by a mechanism that involves recently discovered pathways of AID-dependent but T-cell- and GC-independent SHM [179]. Consistent with the moderate mutational load in their expressed *IGV* genes, MZ B cells contain molecular footprints of past proliferation in an extrafollicular environment [154, 173, 180, 181]. Prenatal studies have lent additional support to the notion that MZ B cells are able to mutagenize *IGV* genes in the absence of GCs and independent of stimulation with antigen [154, 173, 180, 181]. Second, the splenic marginal zone may be involved in induction of *IGV* mutations because it contains foci of proliferating, clonally related B cells that express AID [182, 183]. These B cells make use of a specialized TI pathway of SHM that involves the recognition of commensal antigens presented on neutrophil extracellular traps (NETs) formed by neutrophils in the marginal or perimarginal zone. Neutrophils of this sort have been designated neutrophil B cell helper (N_BH_) cells [183]. Findings that SHM is decreased in MZ B cells of neutropenic patients [183] underline the relevance of N_BH_ cells. TLR signaling must be important for the N_BH_ pathway of SHM because results show that microbial TLR ligands control the number of splenic N_BH_ cells with mutation-inducing activity [183]. There is also direct evidence that TLR ligation in MZ B cell precursors induces SHM [184, 185]. Third, a group of investigators noticed that splenic CD21^+^CD27^+^B220^+^CD23^−^ MZ B cells localize around the follicular B cell zone in an area similar but not identical to that of the marginal zone in mouse and, hence, termed the cells MZ-analog or MZA B cells [186]. They found that MZA B cells highly express TACI and BAFFR and are hyperresponsive to signals that mimic T-cell activation [186]. They also reported that compared to switched MZA B cells, IgM^+^ MZA B cells respond poorly to inducers of plasmacytic differentiation *in vitro* [187]. This is reminiscent of the lymphoplasmacytic phenotype of WM cells, which appear to respond poorly to inducers of plasmacytic differentiation *in vivo*. In sum, a considerable body of circumstantial evidence suggests that MZ B cells contribute to the MYD88-TIRAP-IRAK4-dependent natural effector memory B cell pool under normal steady-state conditions.

### 5.3. IgM^+^IgD^+^ Memory B cells May Be Derived from B1 Cells

A newcomer among human B cell subsets that is still somewhat controversial but potentially important for the MYD88-TIRAP-IRAK4-dependent natural effector memory B cell population is the B1 B-lymphocyte—the long-searched equivalent of the mouse B1 B cell lineage responsible for most of the spontaneous IgM production in mice. The human B1 subset, identified as CD27^+^IgD^+^CD43^+^, displays typical characteristics of “primitive” or “innate-like” B cells, including tonic BCR signaling, low level of spontaneous IgM^+^ plasma cell production, and capacity to drive allogenic T-cell proliferation [188, 189]. B1 B cells perform a variety of biological functions (e.g., antigen presentation and priming of T-cells), but their principal role is the secretion of natural antibodies (prominently including IgM) in the absence of exogenous antigenic stimulation [190]. Natural antibodies are low-affinity, polyreactive immunoglobulins that use *μ* or *α* H chains that typically harbor only a minimal number of somatic mutations and few if any insertions of nontemplated nucleotides at V(D)J junctions [191]. Natural antibodies provide instant defense against invading pathogens, and also prevent autoimmunity by virtue of inducing a noninflammatory, nonimmunogenic clearance pathway for altered self-antigens including apoptotic bodies [192]. Curiously enough, the CD27^+^IgD^+^CD43^+^ subset was recently shown to comprise around 40% of CD27^+^ B cells in adults, a proportion quite similar to the ~50% CD27^+^IgD^+^IgM^+^ cells indicated in Figure 2. This has raised the question as to whether B1 B cells and natural effector B cells are largely overlapping and what the true contribution of MZ B cells to the natural effector memory cell compartment might be. Although it may be appealing from a conceptual point of view to essentially equate circulating B1 B cells with the CD27^+^IgM^+^IgD^+^ compartment [150], this interpretation has been challenged by serious concerns about the effective size of the human B1 B cell population [193, 194]. Be this as it may, the possibility that B1 B cells make a significant contribution to CD27^+^IgM^+^IgD^+^ memory cannot be rejected.

### 5.4. Alternative Sources of IgM^+^IgD^+^ Memory B cells

Despite the arguments in favor of MZ B and B1 B cells presented previously, the occurrence of somatic mutations in *IGV* genes expressed in CD27^+^IgM^+^IgD^+^ memory B cells has fostered an alternative interpretation of the origin of these cells. Just like CD27^+^IgM^+^IgD^−^ memory B cells, it has been proposed that CD27^+^IgM^+^IgD^+^ memory B cells derive from follicular B cells that have prematurely aborted the GC where they underwent moderate levels of SHM but avoided CSR [195]. Although this interpretation does not flat-out reject the theory that MZ B- and B1 B cells constitute separate, GC-independent B cell diversification pathways [196], it questions the role of innate-like B cells in the formation and maintenance of the natural effector memory B cell compartment [197]. To further complicate the picture, it is possible that additional types of innate-like B cells exist that are able to feed the natural effector memory B cell compartment. A case in point is the CD21^low^CD23^−^CD38^low^CD86^hi^ B cell that expresses IgM and IgD with minimal or no mutations and has been repeatedly described to expand under nonphysiological conditions [198]. These cells were first identified in patients with common variable immunodeficiency (CVID), especially in those with splenomegaly and granulomatous disease [199], but later found to be abnormally elevated in peripheral tissues, such as the bronchoalveolar space [198]. In theory, it is easy to imagine that different types of B cells and biological pathways of B cell development overlap to shape the immune repertoire of natural effector memory B cells. Slow, ongoing induction of SHM via TI pathways triggered by signals from commensal microbial products may cooperate with immunization- or infection-dependent stimuli that rely on both extrafollicular TI and follicular TD pathways. The combination of these pathways would ensure the development of a highly diversified repertoire of ready-to-use innate-like natural effector cells [198], some of which may go on to become precursors of WM.

### 5.5. Constitutive Active MYD88 Signaling in WM

No matter what the true origin of MYD88-TIRAP-IRAK4-dependent natural effector memory B cells might be, the finding that these cells are largely dependent on MYD88 signaling provides an intriguing link to the natural history of WM. MYD88 was put at center stage of WM research when pioneering work at the Dana-Farber Cancer Institute, Boston, MA, demonstrated that the great majority of IgM^+^ LPL contains a specific somatic point mutation in the protein-encoding portion of the *MYD88* gene, which replaced a leucine (L) residue at position 265 of *MYD88* with a proline (P) residue. The highly recurrent exchange was uncovered by next-generation whole-genome sequencing and then confirmed using conventional Sanger sequencing [99]. The mutation and the resulting tumor allele are designated L265P and *MYD*88^L265P^, respectively. L265P resides in the TIR domain of the MYD88 protein, which interacts with TIR domains of various membrane receptors involved in innate immune responses, such as TLRs, IL-1R, and IL-18R. The occurrence of the L265P mutation has been confirmed in independent studies, reporting incidence rates in WM patients that range from 70% to detection in all cases [200–203]. Evidence indicates that *MYD*88^L265P^ is a gain-of-function mutation, causing elevated activity of the adaptor protein, MYD88, in TLR and interleukin signaling [204]. Following receptor stimulation, MYD88 is recruited to the activated receptor complex as a homodimer, which then complexes with interleukin-1 receptor-associated kinase (IRAK) 4 to activate IRAK1 and IRAK2 [177, 178]. This leads to IRAK1-dependent phosporylation of tumor necrosis factor receptor–associated factor 6 (TRAF6), which results in nuclear factor *κ*B (NF-*κ*B) activation via I*κ*B*α* phosphorylation. NF-*κ*B is essential for growth and survival of WM cells. Inhibition of MYD88 signaling inhibits L265P-mutated WM cells by blocking I*κ*B*α* phosphorylation and nuclear translocation of transcriptionally active NF-*κ*B dimers [203]. Bruton tyrosine kinase also contributes to MYD88-dependent activation of NF-*κ*B in WM cell that harbors the L265P mutation [203]. It is interesting to note that—in accordance with the distinction of specific “WM genes” and “general B-lymphoma predisposition alleles” outlined in Section 2.3—*MYD*88^L265P^ should be viewed as the latter. Thus, *MYD*88^L265P^ was detected in B-CLL and ABC-type diffuse large B-cell lymphoma and later on also found in primary central nervous system lymphoma and mucosa-associated lymphoid tissue lymphoma [205–209]. Although the oncogenic *MYD*88^L265P^ allele is neither specific for WM nor was it first discovered in patients with WM, in no case does it occur as consistently as in WM. This defines a clear path forward for improving the outcome of WM. 

### 5.6. Key Points and Future Directions

Unlike “IgM-only” IgM^+^IgD^−^ memory B cells, “natural effector” IgM^+^IgD^+^ memory B cells depend on MYD88-IRAK4-TIRAP signaling for development and/or survival. Natural effector B cells are probably derived from MZ and B1 B cells, but opposing views that consider follicular and other types of B cells also exist. Regardless how this puzzle resolves, the disappearance of natural effector cells under conditions of MYD88 deficiency and the strong selection for the *MYD*88^L265P^ gain-of-function allele in WM support the hypothesis that WM may originate from primitive, natural antibody-producing B-lymphocytes that are dependent on MYD88 signaling for homeostatic maintenance under normal conditions but highly susceptible to neoplastic development upon acquisition of enhanced MYD88 signaling due to the L265P mutation. Laboratory mice may lend themselves to testing the possibility that this mutation confers a strong selective advantage for tumor development. Of great relevance to that end is the finding that L265P occurs at a residue that has been invariant (unchanged) over eons of biological evolution. Thus, L265 has been conserved across the animal kingdom, ranging from frogs to rodents all the way to human beings, indicating that this residue is crucial for MYD88 function.

## 6. Recapitulating Human WM in Laboratory Mice

### 6.1. The Laboratory Mouse Is the Premier Model Organism for Research on Human WM

Because of the low incidence, indolent clinical course, and a variety of other reasons, WM has been woefully neglected in the biomedical research arena compared to its closely related but more prevalent or aggressive cousins among mature B cell and plasma cell neoplasms, such as CLL and MM. The logical consequence of persistent underfunding of WM research is the existence of major knowledge gaps in our understanding of the pathophysiology of the disease. Closing these gaps may require the development of good experimental model systems of human WM. The laboratory mouse lends itself to that purpose because it is the undisputed premier model organism for human diseases including cancer. It also serves as the workhorse of cancer genetics. Among the many advantages the laboratory mouse affords is the close genetic relationship to man; that is, virtually all genes in human beings have a homologue with comparable function in mouse. Additionally, methods for manipulating the mouse genome, including gene targeting for “knocking” mouse genes “out” and human genes “in,” have been perfected. This permits us to “humanize” mice such that genetically engineered strains of mice are able to mimic human disease manifestations more accurately than ever before. On the other hand, the inherent challenges of modeling a complex human disease like WM in laboratory mice should not be underestimated. Despite the many strengths of the “mighty mouse,” it is currently not possible to fully recapitulate human WM in laboratory mice, neither with regard to reproducing WM-typical somatic mutations in appropriate target cells (genocopy of human WM) nor with regard to reproducing the complete spectrum of WM-associated clinicopathologic features and laboratory findings (phenocopy of human WM). The sections below will attempt to summarize the efforts of the biomedical research community to model human WM in mice. This can be conceptually divided in “first generation” xenotransplantation models of WM and “second generation” transgenic models. The latter yield WM-like tumors that develop *de novo* (spontaneously) in the presence of a fully functional, innate, and adaptive immune system, similar to tumor development in human beings.

### 6.2. Xenotransplantation Models of WM

**Figure 6:**
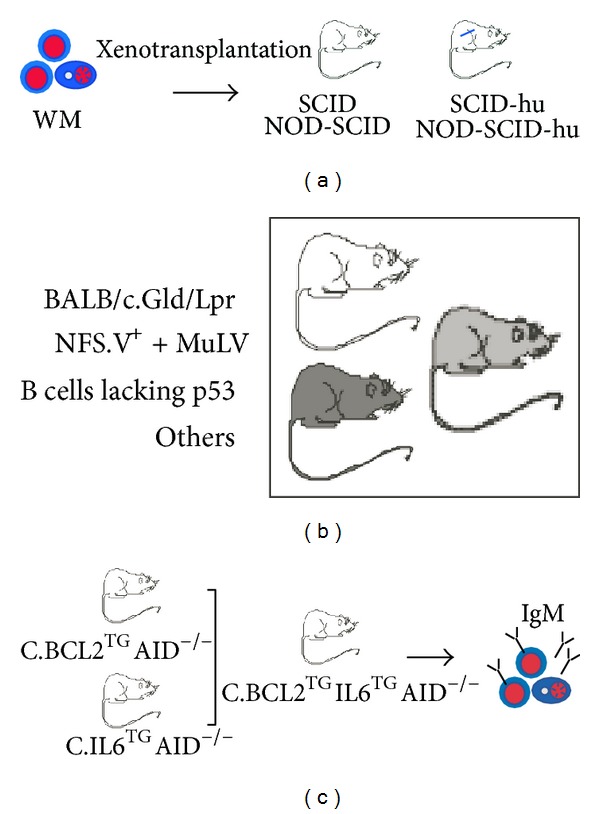
Mouse models of human WM. (a) Xenotransplantation models rely on the propagation of human WM cells in SCID mice that may contain human bone (blue) as a nesting ground for incoming tumor cells. (b) Three examples of strains of mice that are prone to WM-like tumors. Tumor development in NSF.V^+^ mice is dependent on insertional somatic mutagenesis affected by an infectious ecotropic murine leukemia virus. See Section 6.3 for details. (c) Generation of strain C.IL6/BCL2/AID^null^ mice, an IWMF-sponsored project in the laboratory of the author of this paper. The compound transgenic mouse strain combines two independently developed oncogenes, H2-L^d^-IL6 (hereafter called *IL6*) and E*μ*SV-Bcl-2-22 (hereafter called *BCL2*), on the genetic background of AID-deficient BALB/c (c) mice. Strain C is uniquely susceptible to late-stage B cell and plasma cell tumors [219, 220] due to a complex genetic trait that includes hypomorphic (weak efficiency) alleles of genes encoding the cell-cycle inhibitor, p16^INK4a^ [221], and the FKBP12 rapamycin-associated protein, Frap [222], and the absence of the interferon-inducible gene, *Mndal* [223]. The scientific rationale for using the *IL6* transgene is strong and includes the following considerations. IL-6 is a pro-inflammatory cytokine that impacts many blood cancers [224], including Hodgkin lymphoma [225], non-Hodgkin lymphoma [226] and myeloma [227, 228]. In human WM, IL-6 has long been recognized as a major growth, differentiation, and survival factor. In mice, IL-6 is firmly linked to inflammation-dependent plasmacytoma (PCT) induced by i.p. injection of pristane [229]. This treatment provokes the formation of inflammatory granulomas, a rich source of IL-6 at the site of tumor development [230]. Critical evidence for the involvement of IL-6 in inflammation-induced PCT was first obtained in studies with C mice, showing that tumor growth is enhanced by exogenous IL-6 but inhibited by antibodies to IL-6 or its receptor [231]. Subsequent work demonstrated that C mice homozygous for a null allele of *IL6* are resistant to both inflammation-induced PCT [232] and Myc/Raf retrovirus-accelerated PCT [233]. Conversely, C mice carrying the *IL6* transgene develop PCT spontaneously without a requirement peritoneal inflammation [234]. The scientific rationale for using the *BCL2* transgene is equally compelling. Enforced expression of the BCL-2 death repressor [235] increases the apoptotic threshold for tumor precursors because BCL-2 blocks many death signals during normal B cell development [236, 237]; cooperates with oncogenes *in vitro* to promote mitogen-independent cell survival [238]; and accelerates oncogene-driven B-lymphoma *in vivo* [239, 240]. Previous work with *BCL2* transgenic mice and a highly similar transgene [241, 242] showed that BCL-2 selects B cells harboring deregulated oncogenes for tumor development [243, 244]. BCL2 overexpression keeps alive B cells that would normally be eliminated due to developmental problems [245], autoreactivity [246], dysfunctional BCR [247], or illegitimate genetic rearrangements [248]. Loss of AID will modify BCL2/IL6-driven tumor development such that only IgM^+^ tumors can arise; this is because AID is essential for class switch recombination (CSR) [249, 250]. AID is encoded by *Aicda*; it deaminates DNA cytidine to uracil residues, which results in U:G mismatches [251]. Tumor induction studies using AID^−/−^ mice that carry *IL6* or *Bclx* transgenes have been performed [252–254], indicating that the ongoing approach using C.IL6/BCL2/AID^null^ mice should be similarly successful.

The first approach to modeling human WM in laboratory mice relied on *in vivo* propagation of human tumor cells in immunocompromised mice. In these human-mouse xenotransplantation models, fully transformed tumor cells—either obtained from patients with WM or *in vitro* cultures of permanent WM cell lines—are transferred to severe combined immunodeficiency (SCID) mice, which provide the nesting ground for tumor cell engraftment. The SCID mice may harbor subcutaneous implants of human bone (SCID-hu) [210]—or, in the case of the still more severely immunocompromised nonobese diabetic (NOD) SCID strain, intramuscular implants of human bone (NOD-SCID-hu) [211]—to provide the kind of microenvironment (hematopoietic bone marrow) WM cells prefer and are used to (Figure 6(a)). The WSU-WM-SCID model [212, 213], which was used to evaluate the experimental drug XK469 [214], and related models have made and continue to make valuable contributions to the preclinical assessment of WM therapeutics. However, technical and logistic barriers have prevented these models from having significant impact on the process of WM drug discovery; that is, they are not extensively used in preclinical trials and have not gained wide acceptance in the pharmacological industry. Another shortcoming is that, by their very nature, SCID models are not suitable for studies on tumor development and prevention, as this would require experimental model systems in which neoplasms arise spontaneously. Finally, the two cell lines most widely used in the past, WSU-WM and BCWM.1 [215], were later shown to be “nonrepresentative” of WM on the basis of their derivation from EBV-transformed B lymphoblasts [216]. This has questioned the validity of published results on these cell lines and spurred investigators to develop new cell lines that are truly representative of WM. A breakthrough in that regard was the recent establishment of cell line, MWCL-1 [217]. Genetic analysis unambiguously demonstrated the derivation of this cell line from tumor tissue of a patient with WM. MWCL-1 cells produce IgM/*κ* and exhibit an immunophenotype that is fully consistent with WM: CD19^+^CD20^+^CD27^+^CD38^+^CD138^+^. Like many other continuous human cancer cell lines, MWCL-1 cells are deficient for the p53 tumor suppressor: one copy of the p53-encoding gene, *TP53,* is deleted, whereas the second copy is mutated. Importantly, MWCL-1 cells harbor the mutant *MYD*88^L265P^ protein, the hallmark of WM described in Section 5.5. MWCL-1 has not only served as blueprint for generating a valuable additional WM cell line very recently [218], it also has rekindled interest in the research community to employ xenotransplantation models for preclinical studies of new WM drugs.

### 6.3. Transgenic Mouse Models of Human WM

WM-like tumors that arise spontaneously in genetically engineered mice have been described on several occasions in the peer-reviewed cancer literature. Examples include IgM^+^ B cell lymphomas with lymphoplasmacytic features that developed in BALB/c mice deficient in Fas signaling (C.Lpr/Gld) [255, 256], strain NSF.V^+^ mice congenic for ecotropic murine leukemia virus (MuLV) [257, 258], and compound transgenic mice in which the *Trp53*-encoded tumor suppressor, p53, had been specifically inactivated in mature B cells [259] (Figure 6(b)). The strains of mice mentioned previously are available for further improvement, a priority of which may be the introduction of the mutant *MYD*88^L265P^ allele. The potential promise of crossing in *MYD*88^L265P^ has been underlined by a recent study showing that autoantibody production in transgenic mouse models of human systemic lupus erythematosus (SLE) depends on MYD88 [260]. Taking advantage of lupus-prone *MRL*.Fas^lpr^ mice in which MYD88 had been selectively inactivated in B cells, the investigators showed that self-reactive antibodies did not arise when MYD88 signaling was abrogated in B cells [261]. The results suggested that transgenic mice that exhibit elevated MYD88 signaling in B cells due to cell lineage-restricted expression of *MYD*88^L265P^ will be prone to increased autoimmunity followed by formation of WM-like lymphoma. This hypothesis warrants testing. In the author's laboratory, WM-like tumors have repeatedly been observed in mice that carried human *IL6* [234] or *BCL2* [262] transgenes, although the findings were never highlighted in the publications as these were focused on “switched” IgG^+^ or IgA^+^ tumors. The findings led to a research project sponsored by the International Waldenstrom's Macroglobulinemia Foundation (IWMF). The project is aimed at generating compound transgenic IL6/BCL2/AID^null^ mice designed to recapitulate important features of human WM (Figure 6(c)). The rationale for this approach includes our unpublished finding that IL-6 and BCL-2, two key players in human WM (Figure 2), collaborate effectively in neoplastic B cell development in mice. Also included is the expectation that the transfer of these oncogenes to a background that is genetically deficient for AID (activation-induced cytidine deaminase) blocks IL-6/BCL-2-driven tumor development at the IgM^+^ stage. This is because AID is required for CSR. The outcome of this ongoing investigation notwithstanding, there is good reason to be optimistic that designer models of human WM are feasible. Models of this sort will not only permit us to study different stages of tumor progression, including the transition from IgM MGUS to frank WM, but also facilitate the design and testing of new WM interventions.

### 6.4. Key Points and Future Directions

Accurate transgenic mouse models, in which WM-like neoplasms develop with short onset (short tumor latency), high genetic penetrance (high tumor incidence), and a predictable and reproducible tumor pattern, are important research tools for furthering our understanding of the natural history of the disease, particularly with regard to tumor initiation and promotion. Designated mouse models of WM may also be helpful for evaluating new approaches to WM treatment and prevention, including selective small-molecule inhibitors of IRAK and Bruton's tyrosine kinases aimed at dampening MYD88 signaling.

## Abbreviations

AID: Activation-induced cytidine deaminase
*BCL2*: B cell leukemia 2 gene
BCL-2: B cell leukemia 2 oncoprotein
CLL: Chronic lymphocytic leukemia
CSR: Class switch recombination
GC: Germinal center
HSV: Hyperviscosity syndrome
Ig: Immunoglobulin
IgH: Immunoglobulin heavy chain
IgM: Immunoglobulin M
IgV: Immunoglobulin variable gene
*IL6*: Interleukin 6 gene
IL-6: Interleukin 6 gene product (cytokine)
LPL: Lymphoplasmacytic lymphoma
LPC(s): Lymphoplasmacytic cell(s)
MCL: Mantle cell lymphoma
MGUS: Monoclonal gammopathy of undetermined significance
MM: Multiple myeloma
mIgM: Monoclonal immunoglobulin M, IgM paraprotein, IgM spike
MZL: Marginal zone lymphoma
PC(s): Plasma cells
SHM: Somatic hypermutation
SMZL: Splenic marginal zone lymphoma
TI: T-cell independent
TD: T-cell dependent
WM: Waldenström macroglobulinemia.
